# Genome-wide association analysis provides molecular insights into natural variation in watermelon seed size

**DOI:** 10.1093/hr/uhab074

**Published:** 2022-01-19

**Authors:** Chengsheng Gong, Shengjie Zhao, Dongdong Yang, Xuqiang Lu, Muhammad Anees, Nan He, Hongju Zhu, Yong Zhao, Wenge Liu

**Affiliations:** Zhengzhou Fruit Research Institute, Chinese Academy of Agricultural Sciences, Zhengzhou 450009, China

## Abstract

Watermelons used for seed consumption tend to have larger seeds, whereas watermelons used for flesh consumption often require relatively small seeds. Therefore, watermelon seed size has received extensive attention from consumers and breeders. However, the natural variation and genetic mechanism of watermelon seed size remain unclear. In the present study, 100-seed weight, seed hilum length, seed length, seed width, and seed thickness were examined in 197 watermelon accessions. Furthermore, association analysis was performed between seed size traits and high-quality SNP data. The results revealed that there were strong correlations among the five seed traits, and seed enlargement was an important feature during watermelon seed size domestication. The seed-consumed biological species *Citrullus mucosospermus* and the edible seed watermelon *Citrullus lanatus* had significantly larger seeds than the other species. Eleven non-repeating significant SNPs above the threshold line were obtained from GWAS analysis. Four SNPs on chromosome 5 were considered to be closely associated with seed size traits (S5:32250307, S5:32250454, S5:32256177, and S5:32260870) and could be used as potential molecular markers for the breeding of watermelon cultivars with a target seed size. In addition, based on gene annotation information and previous reports, five genes near the four significant SNPs may regulate seed size. qRT-PCR analysis showed that two genes that may be involved in abscisic acid metabolism, *Cla97C05G104360* and *Cla97C05G104380*, may play an important role in regulating watermelon seed size. Our findings provide molecular insights into natural variation in watermelon seed size and valuable information for molecular marker-assisted breeding.

## Introduction

Watermelon is an important Cucurbitaceae crop that is widely grown around the world [1] and is classified into seven biological species, CA (*Citrullus amarus*), CC (*Citrullus colocynthis*), CE (*Citrullus ecirrhosus*), CL_CUL (*C. lanatus* cultivar), CL_LR (*C. lanatus* landrace), CM (*C. mucosospermus*), and CN (*Citrullus naudinianus*) [2, 3]. Notably, natural and human selection have caused significant phenotypic differences in traits such as fruit size and sugar content during the long-term domestication of watermelon [4]. The seed size of watermelon may also have been domesticated. Flesh-consumed watermelons often belong to *C. lanatus*, and relatively smaller seeds can increase the edible part of the watermelon flesh, making cultivated seedless or small-seeded watermelons more popular in the marketplace. By contrast, CM and *C. lanatus* edible-seeded watermelons (CL_ES), which are used for seed consumption, often have larger seeds. A more comprehensive understanding of the natural variation in watermelon seeds is important for breeding cultivars with target seed sizes and may provide insights into the domestication process of different species. However, we currently lack complete data on watermelon seed size in natural populations.

In the plant kingdom, seed size is usually regulated by multiple genes. Previous studies have identified the ubiquitin-proteasome pathway, G-protein signaling, and signaling pathways such as mitogen-activated protein kinase (MAPK) signaling as the three main pathways that affect seed size by regulating the size of maternal tissue [5]. In addition, transcription factors such as BS1, AP2, and WRKY [6, 7], genes in the HAIKU (IKU) pathway, and plant hormones such as abscisic acid (ABA), brassinosteroids, and auxin are also thought to play essential roles in the regulation of seed size [8–10]. At present, some progress has been made in understanding watermelon seed traits. Li et al. [11] found that the black seed coat of watermelon was controlled by a single dominant gene and identified the candidate gene *ClCS1* for seed coat color regulation by fine-mapping. Li et al. [12] identified three candidate genes by fine mapping based on the main QTL *qSS6* for thousand-seed weight, seed length, and seed width; these included homologs of the rice seed size regulator *SRS3*. Wang et al. [5] obtained the key gene *ClBG1* that regulates seed size in watermelon; knockout of this gene reduced ABA content, leading to smaller watermelon seeds and increased germination. It is worth noting that ABA is a key hormone that regulates seed germination. Previous studies have shown that ABA content plays an important role in regulating watermelon seeds [5]. Therefore, genes that affect the accumulation of ABA, such as *PP2C* [13, 14], may also affect seed size by regulating ABA content.

**Table 1:** Descriptive statistics of five seed traits in watermelon

**Trait**	**Mean**	**Maximum**	**Minimum**	**SD**	**CV (%)**	**Kurtosis**	**Skewness**
**100SWT-2019**	7.93	23.50	0.90	4.28	53.97	3.49	1.03
**100SWT-2020**	7.51	21.44	0.84	4.02	53.59	3.62	1.09
**SHL-2019**	3.34	6.23	1.35	0.80	23.97	3.23	0.18
**SHL-2020**	3.39	6.46	1.30	0.83	24.46	3.39	0.23
**SL-2019**	10.69	17.98	4.56	2.70	25.23	2.37	0.45
**SL-2020**	10.53	18.51	4.13	2.63	25.01	2.73	0.57
**SWD-2019**	6.59	12.47	3.20	1.62	24.56	3.40	0.74
**SWD-2020**	6.47	11.06	2.63	1.57	24.30	3.26	0.74
**STH-2019**	2.26	3.78	1.64	0.31	13.76	5.58	1.02
**STH-2020**	2.22	3.91	1.60	0.31	14.01	6.79	1.24

Note: SD, standard deviation.

CV, coefficient of variation.

Genome-Wide Association Study (GWAS) is a method for analyzing genetic polymorphisms in multiple individuals by combining them with phenotypic traits in the natural population. Currently, association analysis is often performed with the GEMMA [15], Factored Spectrally Transformed Linear Mixed Models (FaST-LMM) [16], and Efficient Mixed Model Association eXpedited (EMMAX) [17] genome-wide association methods. Significant progress has been made in understanding the seed sizes of rice, *Brassica napus*, and other plants using GWAS [18–20]. GWAS has also enabled important progress in understanding the genetic basis of watermelon agronomic traits. Guo et al. [4] resequenced 414 watermelon accessions and identified key regulatory genes for sugar content, fruit color, seed coat color, and other important traits using GWAS. Dou et al. [21] combined the results of GWAS and BSA and obtained the key gene *ClFS1* that regulates watermelon fruit shape. GWAS analysis provides a useful tool for the genetic study of important agronomic traits. However, there are no research reports on obtaining SNPs and candidate genes closely related to seed size traits using this method.

Understanding the natural variation in seed size may increase our understanding of watermelon domestication and improvement, and genetic research will provide molecular insights for the selection of new cultivars with target seed traits. In the present study, five seed size traits, 100-seed weight (100 SWT), seed hilum length (SHL), seed length (SL), seed width (SWD), and seed thickness (STH), were measured and analyzed for 197 watermelon accessions in 2019 and 2020. The significant SNP loci linked to seed traits obtained by GWAS can be used as potential molecular markers for molecular marker-assisted breeding. Furthermore, candidate genes near SNP loci were analyzed by quantitative real-time PCR (qRT-PCR). Taken together, our findings provide data support for the genetic basis of watermelon seed size.

## Results

### Natural variation in seed size traits of 197 watermelon accessions

The natural variation in five seed size traits in 2019 and 2020 was evaluated using the statistics of seven indicators: maximum, minimum, mean, standard deviation, coefficient of variation, kurtosis, and skewness. The results showed that there were no significant differences in the values of these indexes by comparing the data from the two years (Table 1). The correlation of the five seed traits between 2019 and 2020 was > 94%, indicating that the phenotypic data for the two years showed a good correlation. In addition, phenotypic coefficient of variation analysis showed that 100 SWT had the highest variability, i.e. 53.97% and 53.59% in 2019 and 2020, much higher than that of SHL (23.97%, 24.46%), SL (25.23%, 25.01%), SWD (24.56%, 24.30%), and STH (13.76%, 14.01%). The skewness values of all traits were > 0, and the kurtosis values of all traits except SL were > 3. Thus, these five traits were quantitative traits that followed a normal distribution in the 197 watermelon accessions. In short, the five seed traits were quantitative traits with significant phenotypic variation and strong correlations between the two years.

**Figure 1:**
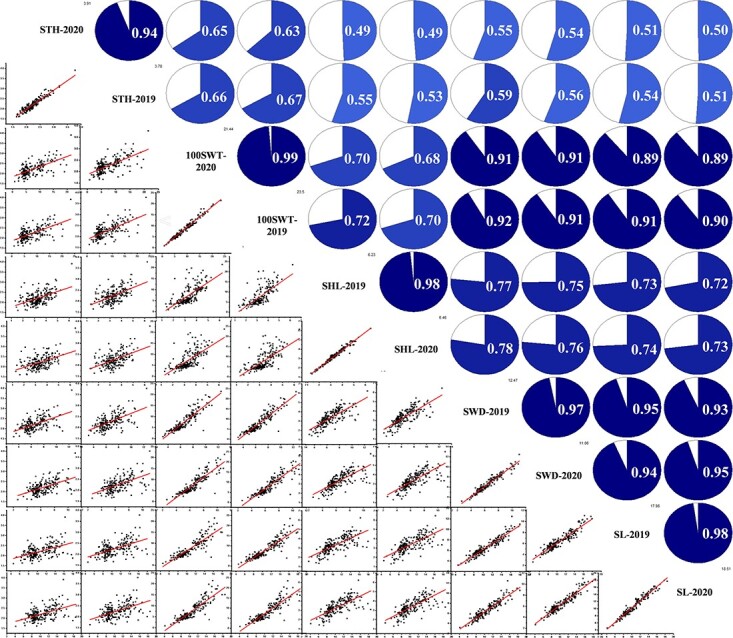
Correlation analysis of five seed traits of 197 watermelon accessions in 2019 and 2020. The correlation values and correlation pie charts are displayed in the upper triangle, and the scatter plots between pairs of seed traits are shown in the lower triangle. 100-seed weight (100 SWT), seed hilum length (SHL), seed length (SL), seed width (SWD), and seed thickness (STH).

The 100 SWT is an important indicator of seed size because it is usually a combination of SL, SWD, SHL, and STH. To better understand the relationships among the five seed size traits and their contributions to seed size formation, correlation analyses were performed on data from the two years (Fig. 1). In 2019, the correlations between 100 SWT and SL/SWD exceeded 90%, whereas its correlations with SHL (72%) and STH (67%) were relatively low. In addition, the correlation between SL and SWD was the highest (95%), the correlations between STH and the other four traits were < 70%, and the correlations between SHL and the other four traits were < 80%. At the same time, there were no significant differences between the correlation values in 2020 and 2019 (Fig. 1). These data indicated that SL and SWD were more likely to determine seed size variability than STH and SHL.

### Obvious phenotypic differences in seed size traits among different biological species

Statistics for the ten accessions with the largest and smallest 100 SWT in the two years showed that the 100 SWT of the largest ten accessions exceeded 15.5 g and that NingXianXiGua, PI179240, PI532722, PI560014, DaBanGuaZi, DaBanHongZiGua, DengKouZiGua, and GaolanZiGua were among the largest 100 SWT accessions in both 2019 and 2020. Among these eight accessions, GaolanZiGua had the largest 100 SWT, exceeding 20 g in 2019 and 2020. It is worth noting that NingXianXiGua belongs to CL; PI179240, PI532722, and PI560014 belong to CM; and the other four accessions belong to CL_ES. Among the ten accessions with the smallest 100 SWT in 2019 and 2020, TOMATOSEED, SuXianXiaoZi, 9904, ZhuXiaoHeiXiaoZi, XiaoZiMiBao, ZXG1702, and PI386015 were common to both years. Among these seven accessions, TOMATOSEED had the smallest seeds, and its 100 SWT was <1 g. Interestingly, the first five accessions belong to CL, and the other two accessions belong to CC. Through the analysis of extreme watermelon seed size, we found that there may be significant differences in seed size among different species of watermelon.

**Figure 2:**
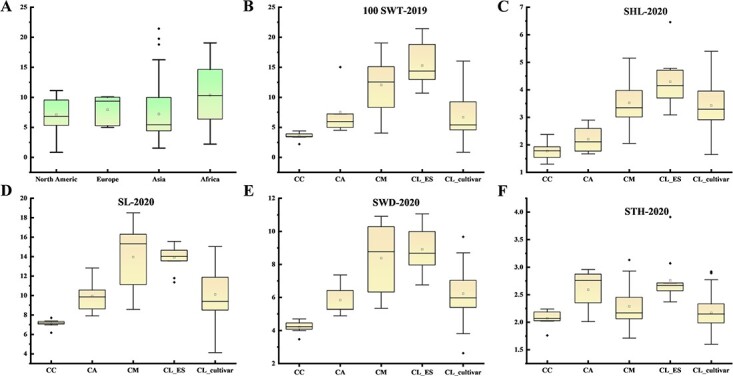
Statistical analysis of five seed size traits in different types of watermelon in 2020. (**A**) Box plot of 100 SWT in watermelon from different geographic origins. (**B–F**) Box plots of 100 SWT (g), SHL (mm), SL (mm), SWD (mm), and STH (mm) in different types of watermelon. CC: *Citrullus colocynthis*; CA: *Citrullus amarus*; CM: *Citrullus mucosospermus*; CL_ES: *Citrullus lanatus* edible seed watermelon; CL: *Citrullus lanatus*.

Among the 197 watermelon accessions, 18 were from North America, 20 from Africa, 5 from Europe, and 152 from Asia. The results showed no significant differences in 100 SWT among accessions from different regions (Fig. 2A). These accessions included 8 CC, 5 CA, 17 CM, 11 CL_ES, and 156 CL (**Table S1****)**. Analysis of the 100 SWT of different watermelon species in 2020 showed that CC had the lowest seed weight, with an average 100 SWT of 3.52 g; CL_ES had the highest seed weight, with an average 100 SWT of 15.28 g; and CA, CM, and CL had average 100 SWTs of 7.54 g, 12.60 g, and 6.67 g (Fig. 2B). Similarly, SHL, SL, and SWD showed trends similar to those in 100 SWT among the different watermelon species (Fig. 2C–E). However, for STH, CA and CL_ES had thicker seeds, whereas CC, CM, and CL had thinner seeds (Fig. 2F). In addition, there were no significant differences in these five seed traits between 2019 and 2020 (Fig. 2**,**Fig. S1). These results showed that the five seed traits were smallest in CC; seeds of CA were relatively smaller and thicker, unlike those of CM; and CL_ES had the largest seed weight and did not differ significantly from CM, consistent with the fact that these two types of watermelon are used mainly for seed consumption. CL showed a large degree of seed variation in the natural population. In summary, the significant differences in seed size between different species may be related to the long-term domestication and artificial selection of watermelon.

### GWAS analysis of watermelon seed size traits

Principal component analysis was performed on the high-quality SNP data from 197 watermelon accessions. The results showed that the first two principal components, PC1 (61.86%) and PC2 (6.35%), could divide the watermelons into five groups, and the group structure was consistent with the phenotypic data analysis of seed size traits, indicating that the accessions we selected were reasonable (Fig. S2).

**Figure 3:**
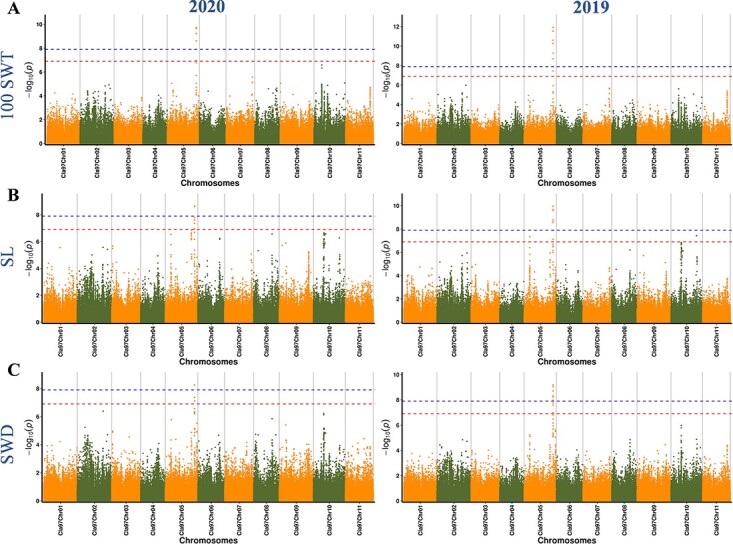
Manhattan plots of GWAS for 100 SWT (**A**), SL (**B**), and SWD (**C**) in 2020 and 2019. The x-axis represents the position along a chromosome, the y-axis represents −log_10_(*P*-value), and each point is the −log_10_(*P*-value) of a SNP locus. The blue and red dotted lines parallel to the x-axis represent the two significance thresholds.

To further establish the association between SNPs and seed size traits in the 197 watermelon accessions, we used the two algorithms of EMMAX and Fast-LMM for association analysis. The results showed that no significant SNP loci were associated with SHL and STH, whereas SNP loci were significantly correlated with the three seed traits, 100 SWT, SL, and SWD. Moreover, the consistency between the observed and expected *P* values was evaluated by a QQ plot, effectively controlling the generation of false positives (Fig. S3). In 2020 and 2019, 5 and 7 significant SNP loci for 100 SWT were identified by EMMAX association analysis; 5 and 10 significant SNPs were identified for SL; and 3 and 9 significant SNPs were identified for SWD (Fig. 3**,****Table S2**). A total of 11 non-repeated significant SNPs were identified on chromosome 5 and chromosome 10. Specifically, S5:32250307 (SNP locus 32 250 307 on chromosome 5), S5:32256332, and S5:32252937 were all associated with three traits in two years. S5:32250454 and S5:32254669, were significantly associated with all traits in the association analysis except for SWD in 2020. S5:32251321 and S5:32256177 were significantly associated with three seed traits in 2019. S5:32180774 was associated only with SL and SWD in 2019. In addition, S10:28482105 and S5:6272374 were additional SNPs identified for SL in 2019, and S5:32260870 was only associated with SWD in 2019. In addition, the Fast-LMM algorithm also produced similar location results on chromosome 5, indicating the reliability of our analysis results (Fig. S4). The 11 non-repeating SNPs that exceeded the thresholds and were significantly associated with three seed size traits will be further analyzed to explore whether they can be used as potential molecular markers.

### Analysis of significant SNPs associated with seed size traits

**Figure 4:**
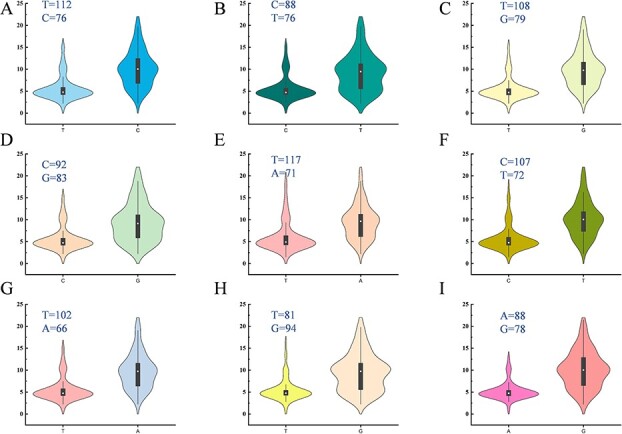
Violin plots diagrams of significant SNP loci for 100 SWT at different bases in 2020. (**A–I**) show 100 SWT in different accessions under nine significant SNP loci, S5:32250307, S5:32256332, S5:32250454, S5:32252937, S5:32254669, S5:32251321, S5:32256177, S5:32180774, and S5:32260870. The black portion of each plot represents the range of 25%–75%, and the white circle represents the median.

The analysis of eleven significant SNPs correlated with seed traits showed that, for SNP locus S10:28482105 on chromosome 10, the average 100 SWT was 7.47 g for base T (173 accessions) and 7.58 g for base C (19 accessions), and this difference was not significant. For locus S5:6272374 on chromosome 5, there was also no significant difference in 100 SWT between the different bases. For the other nine significant SNPs on chromosome 5, seed size traits differed significantly for the different bases (significance level: P < 0.05, **Table S3**). For the most significant 100 SWT and SL SNP locus, S5:32250307, 112 accessions contained base T, with an average 100 SWT of 5.66 g, and 76 accessions contained base C, with an average 100 SWT of 10.17 g (Fig. 4A). The other eight significant SNPs on chromosome 5 also showed significant differences in 100 SWT with different bases (Fig. 4B–I). In addition, these significant differences associated with different SNP loci also existed in SL and SWD (Fig. S5 and Fig. S6). It can be clearly observed that seeds with the mutant SNP bases were significantly larger than those with the reference bases. Previous analysis showed that CC and CA have smaller seeds, and combined with our existing results, we used two criteria to screen for SNPs that were more strongly associated with seed size. The first criterion was that 100 SWT exceed 15.4 g in 2019 and 17 g in 2020 under the mutant base; the second was that CC and CA, with smaller seeds, should contain the reference base. According to the above criteria, we screened out four SNP loci that were significantly related to seed traits, S5:32250307, S5:32250454, S5:32256177, and S5:32260870, which could be used as potential molecular markers for watermelon seed size screening.

### Prediction of candidate genes near significant SNPs

Genes closer to significant SNPs are more likely to be candidate genes for the regulation of target traits. Therefore, combining the LD decay analysis results from a previous report [4], we further analyzed the 100-kb upstream and downstream intervals (S5:32150307–S5:32360870) around the four significant SNPs. Combined with the LD block analysis result, candidate genes were predicted to be in this interval (Fig. S7). In this interval, there were 31 genes (Table 2). Interestingly, based on gene annotations and previous reports on the regulation of seed size, five genes in the upstream and downstream 30-kb interval of the most significant SNP locus S5:32250307 may be key genes for the regulation of watermelon seed size. Specifically, the F-box protein is related to organ size [22], and the protein kinase and zinc finger protein have a regulatory effect on rice seed size [23–25]. In addition, PP2C and chaperone proteins play an important role in ABA formation [13, 14, 26], and ABA is involved in the development of watermelon seeds. Therefore, we assumed that these five genes, i.e. *Cla97C05G104340*, *Cla97C05G104350*, *Cla97C05G104360*, *Cla97C05G104380*, and *Cla97C05G104390*, may be related to watermelon seed size. The two SNPs S5:32250307 and S5:32250454 were located in the promoter region of *Cla97C05G104380*.

**Table 2:** Annotation information for genes that may regulate watermelon seed size in the candidate interval.

**Gene ID**	**Location**	**Annotation**
Cla97C05G104240	Cla97Chr05 : 32152237 .. 32154790 (-)	Lipid phosphate phosphatase 2-like
Cla97C05G104250	Cla97Chr05 : 32169017 .. 32171280 (+)	Octicosapeptide/Phox/Bem1p family protein, putative
Cla97C05G104260	Cla97Chr05 : 32171937 .. 32172218 (+)	DNA polymerase
Cla97C05G104270	Cla97Chr05 : 32174404 .. 32180497 (-)	Elongator complex protein 2
Cla97C05G104280	Cla97Chr05 : 32187371 .. 32189619 (+)	Auxin-responsive protein
Cla97C05G104290	Cla97Chr05 : 32190749 .. 32192979 (-)	Receptor-like kinase
Cla97C05G104300	Cla97Chr05 : 32200396 .. 32200617 (-)	Unknown protein
Cla97C05G104310	Cla97Chr05 : 32212680 .. 32215122 (-)	Fiber protein Fb34
Cla97C05G104320	Cla97Chr05 : 32212680 .. 32215122 (-)	Protein of unknown function (DUF789)
Cla97C05G104330	Cla97Chr05 : 32215162 .. 32215633 (-)	Unknown protein
Cla97C05G104340	Cla97Chr05 : 32219731 .. 32220987(-)	F-box protein
Cla97C05G104350	Cla97Chr05 : 32226196 .. 32231527 (+)	Receptor kinase 3
Cla97C05G104360	Cla97Chr05 : 32238521 .. 32241223 (+)	Phosphatase 2C family protein
Cla97C05G104370	Cla97Chr05 : 32243537 .. 32244053 (+)	Unknown protein
Cla97C05G104380	Cla97Chr05 : 32250484 .. 32254215 (+)	Chaperone protein dnaJ 15
Cla97C05G104390	Cla97Chr05 : 32261955 .. 32262626 (+)	Zinc finger protein 6
Cla97C05G104400	Cla97Chr05 : 32272697 .. 32273092(-)	Cytosolic phospholipase A2
Cla97C05G104410	Cla97Chr05 : 32281966 .. 32283184 (+)	DUF1639 family protein
Cla97C05G104420	Cla97Chr05 : 32285161 .. 32289598 (+)	Acyl-acyl carrier protein thioesterase ATL3, chloroplastic-like
Cla97C05G104430	Cla97Chr05 : 32292841 .. 32293998 (+)	Universal stress protein A
Cla97C05G104440	Cla97Chr05 : 32294842 .. 32296421 (-)	Endonuclease 2
Cla97C05G104450	Cla97Chr05 : 32308050 .. 32309003 (+)	Transmembrane protein, putative
Cla97C05G104460	Cla97Chr05 : 32312666 .. 32313112 (-)	VQ motif-containing protein
Cla97C05G104470	Cla97Chr05 : 32326516 .. 32327544 (-)	Adenylate isopentenyltransferase-like
Cla97C05G104480	Cla97Chr05 : 32338449 .. 32340878 (+)	50S ribosomal protein L15, putative
Cla97C05G104490	Cla97Chr05 : 32341944 .. 32342282 (+)	Unknown protein
Cla97C05G104500	Cla97Chr05 : 32347803 .. 32348087 (-)	Unknown protein
Cla97C05G104510	Cla97Chr05 : 32349038 .. 32349789 (+)	1-deoxy-D-xylulose-5-phosphate synthase 2
Cla97C05G104520	Cla97Chr05 : 32349874 .. 32350638 (+)	Unknown protein
Cla97C05G104530	Cla97Chr05 : 32354081 .. 32354846 (-)	Ribulose bisphosphate carboxylase small chain
Cla97C05G104540	Cla97Chr05 : 32354894 .. 32355681 (-)	Unknown protein

### qRT-PCR analysis of candidate genes in six watermelon accessions

**Figure 5:**
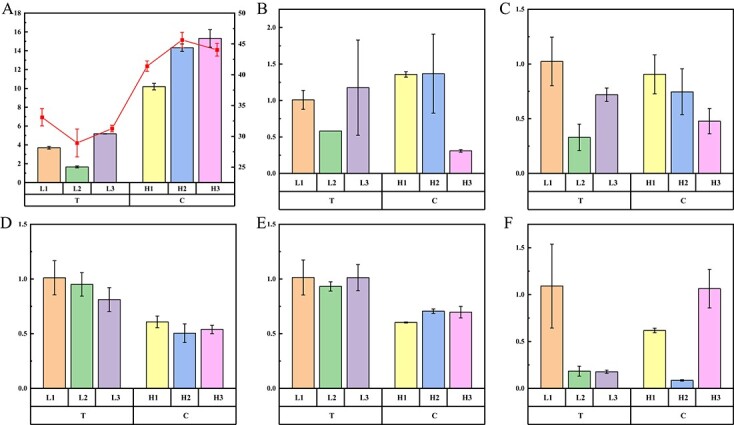
Seed weight (g), ABA content (μg/L) and the expression levels of five candidate genes. (**A**) The bar graph represents seed weight, and the red points represent the ABA content. Gene expression levels of *Cla97C05G104340* (**B**), *Cla97C05G104350* (**C**), *Cla97C05G104360* (**D**), *Cla97C05G104380* (**E**), and *Cla97C05G104390* (**F**) in watermelon seeds at 34 DAP. L1, L2, L3, H1, H2, and H3 represent the accessions XiangXiaoGua, SuXianXiaoZi, XiaoHongYu, HeTaoPi, JiZhuaGua, and NingXiaHongZiGua, respectively. T and C are the reference and mutant bases at SNP locus S5:32250307 on chromosome 5.

RNA was extracted from seeds of six watermelon accessions for qRT-PCR analysis to verify the expression levels of candidate genes. The base at the most significant SNP locus S5: 32250307 was T in the three accessions XiangXiaoGua, SuXianXiaoZi, and XiaoHongYu, and their average 100 SWTs were 3.71 g, 1.66 g, and 5.18 g, respectively. HeTaoPi, JiZhuaGua, and NingXiaHongZiGua contain the mutant base C at this locus, and their average 100 SWTs were 10.19 g, 14.33 g, and 15.31 g, respectively (Fig. 5A). Based on the qRT-PCR results, we found that the expression levels of *Cla97C05G104340*, *Cla97C05G104350*, and *Cla97C05G104390* in watermelon seeds did not show obvious changes or differences between accessions with the different bases at 34 days after pollination (DAP) (Figs. 5B, 5C, 5F). By contrast, *Cla97C05G104360* and *Cla97C05G104380* showed obvious expression differences between the different base backgrounds at 34 DAP (Figs. 5D, 5E). In addition, *Cla97C05G104360* and *Cla97C05G104380* had relatively higher gene expression in the three smaller-seeded materials at 20 DAP (Fig. S8), and similar gene expression results were also found during watermelon flesh development (Fig. S9). As in other plants, members of these two gene families are thought to negatively regulate ABA levels. It has also been confirmed that when *CLBG1*, a gene regulating seed size, was knocked out, seeds became smaller and ABA content decreased. Therefore, we hypothesized that *Cla97C05G104360* and *Cla97C05G104380* may negatively regulate ABA content in watermelon seeds and affect watermelon seed size. In addition, the ABA content of the six cultivars was determined, and the larger seeds had a relatively higher ABA content (Fig. 5A), consistent with the hypothesis that the two genes may negatively regulate ABA content.

## Discussion

### Seed enlargement was an important characteristic of watermelon domestication

Natural variation in crops is generated by wild ancestral plants under natural and human selection, and a full understanding of crop phenotypic variation and the domestication process can help us more effectively improve the diversity of genetic resources [27]. The wild watermelon has bitter, pale-colored, and non-sweet flesh, whereas the cultivated watermelon has non-bitter, well-colored, and sweet flesh [28]. These differences in traits are the result of long-term selection. Among crops, some of the most common domesticated traits in the natural population include decreased seed dormancy and seed enlargement [29], which has also been confirmed in our research. Compared with other types of watermelon, CC and CA had significantly smaller seeds. Specifically, as the ancestor, CC had the smallest seeds. CA had larger seeds than CC, and its STH was thicker than that of the other types, which may be an important reason why wild-type watermelon seeds are difficult to sprout. CM is distributed in Western Africa [2], and CL_ES (belonging to CL–LR) is distributed mainly in China. These two types of watermelon are used mainly for seed consumption. The average SL and SWD of CM were the largest, while with smaller STH; the 100 SWT of CM was smaller than that of CL_ES. The seeds of CL (*C. lanatus* cultivar and *C. lanatus* landrace) showed a large degree of variation. CL seeds had a greater degree of variation, but their average 100 SWT was similar to that of CA, which may be determined by the characteristics of this type of species (*i.e.* flesh-consumed watermelon should have smaller seeds). It is worth noting that in CM and CL_LR (including CL–ES) with larger seeds, linkage disequilibrium decayed to the maximum value within the corresponding distance of about 100 kb, significantly higher than CC and CA [4]. In general, seed enlargement is an important feature of watermelon in the process of domestication, and the seed size characteristics of CM, CL_ES, and CL were mainly determined by seed consumption or fruit consumption.

### GWAS analysis provided potential SNP molecular markers for watermelon seed size traits

Genes are the root cause of differences in traits and provide strong support for research to improve crop yield and quality. For example, overexpression of *zmm28* in maize significantly increases its yield [30], and the monoterpene synthase gene cluster is involved in the formation of carotene flavor [31]. BSA and genetic mapping are common methods of gene fine mapping. Xue et al. identified the candidate interval for red pear skin by BSA and developed available molecular markers [32], and important QTLs regulating watermelon seed size were found on chromosomes 2, 6, 8, and others [33, 34]. However, BSA and genetic mapping tend to require a long time for the establishment of a segregating population. GWAS provides an effective tool for fast and accurate gene mapping of target traits. Guo et al. [35] used GWAS analysis to identify significant SNPs related to grape fruit traits that may be used in molecular marker-assisted breeding. The development and utilization of molecular markers have greatly improved the efficiency of breeding. In the current study, four SNP loci significantly correlated with 100 SWT, SL, and SWD were identified, S5:32250307, S5:32250454, S5:32256177, and S5:32260870. These four significant SNPs provide the first potential molecular markers for seed traits in natural populations.

### Two candidate genes may affect watermelon seed size by regulating ABA content

Based on gene annotation information and previous reports, five genes may regulate watermelon seed size. Specifically, gene annotation information indicates that *Cla97C05G104340* is an F-box protein; in Arabidopsis, the F-box protein STERILE APETALA (SAP)/SUPPRESSOR OF DA1 (SOD3) affects the stability of the transcriptional regulatory factors Peapods (PPDs), which in turn regulate organ size [22]. *Cla97C05G104350* contains a conserved protein kinase domain, STKC_IRAK. In a previous study, non-synonymous mutations in the exon of *BAK1*, an important gene with this conserved domain, resulted in changes in rice grain size [23]. *Cla97C05G104390* is a zinc finger protein with a conserved C2H2 domain. A C2H2 zinc finger protein is thought to affect the development of spikelets in rice, thus affecting seed size and yield [24, 25]. ABA is an important hormone whose content affects seed size in plants such as watermelon [5]. Gene annotation information indicates that *Cla97C05G104360* encodes a PP2C family protein. PP2Cs have been shown to negatively regulate ABA content in previous studies [13, 14], and a study also found PP2C regulation of seed size in soybean. *Cla97C05G104380* encodes a chaperone protein dnaJ gene. In Arabidopsis, ATJ3 (chaperone, dnaJ homolog 3) inhibits the activity of PKS5 (Protein Kinase5) [26], and *PKS5* (SOS2-LIKE PROTEIN KINASE5) is an important gene involved in ABA responses [36], suggesting that dnaJ may affect ABA content through negative regulation in plants. The seeds of six watermelon accessions with different base backgrounds were used for qRT-PCR analysis. *Cla97C05G104360* and *Cla97C05G104380* showed obvious differences in gene expression among different types of watermelon, and these two genes may be closely related to the accumulation of ABA. In previous studies on watermelon seeds, knockout of the gene *CLBG1* resulted in lower ABA content and smaller seeds [5], and we therefore speculate that these two genes may be candidate genes for the regulation of watermelon seed size. It is worth noting that seed size is a complex quantitative trait, and we need combine more experiments on candidate genes with retrograde validation. Specifically, in the next step, we will perform further laboratory work to verify the functions of the candidate genes and construct isolated population validation experiments to further verify their possible key roles in the regulation of watermelon seed size. Overall, our study provides potential molecular markers for gene mapping of watermelon seeds and documents the variation among watermelon seeds in natural populations.

## Materials and methods

### Plant materials and cultivation management

A total of 197 watermelon accessions were sequenced and used in this experiment. The materials were collected from the National Mid-term Genebank for Watermelon and Melon, the Zhengzhou Fruit Research Institute, and the Chinese Academy of Agricultural Sciences (Zhengzhou, Henan, China). The 197 watermelon accessions were planted in Xinxiang (Henan, China) in 2019 and in Zhongmu (Zhengzhou, Henan, China) in 2020. Divided by geographical location, these germplasm resources were mainly from North America, Africa, Europe, and Asia. The classification of different species of accessions was based mainly on the previous report of Guo et al. [4]. The accessions were divided into five types: CC and CA are wild-type watermelons; CM and CL_ES are often used for seed consumption and were classified separately; and CL_CUL and CL_LR are closely related and therefore classified as CL.

In addition, six accessions used for qRT-PCR analysis were planted in Xinxiang in the spring of 2021, and watermelon seeds were sampled at 20 DAP and 34 DAP. The watermelon plants were planted with double pruning. The dates of sowing and transplant to the field were selected based on local climatic conditions, and a randomized complete block design (RCBD) was used with a plant spacing of 0.8 m and a row spacing of 1.5 m. Agronomic practices, including fertilization, irrigation, and pest control, remained consistent in both environments. Pollination was performed by self-crossing, and only one fruit was grown on each plant. The watermelons were harvested based on the characteristics of the different accessions. Fresh seeds were washed and placed in breathable gauze bags. After drying, the dried seeds were used for further phenotypic measurements.

### Measurement of seed size traits

Five seed size traits were determined manually, including 100-seed weight (100 SWT), seed hilum length (SHL), seed length (SL), seed width (SWD), and seed thickness (STH). Seeds with good growth were selected from each independent watermelon to determine the 100-seed weight. Three watermelons were measured as three biological replicates, and measurements were performed with an electronic balance (JA2003, Soptop). Seeds with relatively uniform appearance and good growth were selected for the determination of SHL, SL, SWD, and STH. After seeds were selected from each watermelon, a digital vernier caliper (AIRAJ) was used to measure and record the four agronomic traits. The results from different watermelons of the same cultivar were averaged for further analysis.

### Statistics and analysis of phenotypic data

The maximum, minimum, average, variance, standard deviation, and variation of the five seed traits in 197 watermelon accessions were calculated using Microsoft Excel 2020. To further clarify the overall distribution of the five traits in the natural population, two important indicators, kurtosis and skewness, were also calculated using the following formulas:

$$ K=\frac{\sum_{i=1}^n{\left({x}_i-\overline{x}\right)}^4}{\left(n-1\right){S}^4} $$

$$ g=\frac{\sum_{i=1}^n{\left({x}_i-\overline{x}\right)}^3}{\left(n-1\right){S}^3} $$

where *K* is kurtosis, *g* is skewness, *x*_i_ is the *i*th sample, *n* is the total number of samples, and *S* is the standard deviation.

Correlations between each pair of seed traits were calculated using the default statistical method of Origin software (Origin 2021). The correlation analysis of different seed size traits was visualized using the corrplot R package.

### SNP acquisition discovery and GWAS analysis

Of the 197 watermelon accessions used for GWAS analysis, 163 were sequenced in previous research reports (WM in Table S1) [4], and the genome library construction, sequencing, and analysis methods for the other 34 accessions were based on previous research methods (R in Table S1). SNP data with variation were also obtained based on a previous analysis method [4] and filtered based on secondary allele frequency (MAF: 0.05) and site integrity (INT: 0.8) to obtain highly consistent SNPs for further analysis. To evaluate whether the selected population was suitable for GWAS analysis, principal component analysis (PCA) was performed based on SNP data from the different types of species, and EIGENSOFT [37] software was used to obtain the result of sample clustering. EMMAX [15] and FaST-LMM [16] were used to analyze the five seed size traits for GWAS analysis. The QQ and Manhattan plots were drawn using R software. The significance threshold was calculated using a Bonferroni correction, and −log10 (*P*) 0.1/ Ne (Ne = effective SNP number) and −log10 (*P*) 0.01/Ne were set as the two threshold lines for screening significant SNPs. Based on the characteristics of seed size traits and previous LD decay results, we selected the 100-kb interval upstream and downstream of the significant SNP as the candidate interval, and we performed LD block analysis to increase the probability of the candidate interval. The *C. lanatus* subsp. *vulgaris* cv. 97103 V2 reference genome (http://cucurbitgenomics.org/organism/21) was used for related analysis.

### RNA extraction and quantitative real-time PCR

The seeds of the six watermelon accessions were fully developed and had attained their largest size at 34 DAP, here, the seeds at 20 DAP and 30 DAP were sampled and used for subsequent qRT-PCR analysis. Fresh seeds from the same watermelon were sampled, rapidly quick-frozen in liquid nitrogen, and stored at −80°C. The seeds were ground into powder in liquid nitrogen for RNA extraction. Total RNA extraction was performed according to the instructions of the RNA isolation kit (Huayueyang Biotechnologies, China). cDNA was synthesized using 1 μg RNA as a template according to the instructions of NovoScript Plus All-in-one 1^st^ Strand cDNA Synthesis SuperMix (Novoprotein, China). Gene sequences were obtained based on the Cucurbitaceae genome database (http://www.icugi.org), and *ClCAC* (Gene ID: *Cla97C09G174930*) was used as the reference gene for qRT-PCR. Primers for candidate genes were designed as shown in **Table S4****.** Expression levels of candidate genes were measured on a LightCycler 480 RT-PCR system (Roche, Switzerland). The 20-μL reaction system was constructed according to the instructions of the SYBR Green Real-Time PCR mix (Toyobo, Japan). The main parameters of the program were preheating at 95°C for 5 min and 45 cycles of 95°C for 10 s, 56°C for 30 s, and 72°C for 30 s. To obtain the relative expression levels of the genes, the original data were analyzed by the 2^−ΔΔCT^ method [38].

### Determination of ABA contents in watermelon seeds

The seeds of six watermelon accessions collected at 34 DAP were fully powdered in liquid nitrogen, and 0.2 g–0.5 g of seed powder was accurately weighed into a collection tube. PBS solution (pH 7.3) was added at a weight (g) to volume (ml) ratio of 1:9, and the mixture was placed in a refrigerator at 4°C for 2 h after full shock. After centrifuging at 4°C for 20 min with 3000 r/min (5810R, Eppendorf), the supernatant was collected for testing. Standards were diluted gradually to 300 μg/L, 150 μg/L, 75 μg/L, 37.5 μg/L, and 18.75 μg/L, and the ABA content of the samples was determined following the instructions of the plant ABA ELISA kit (www.mmbio.cn). The standard curve formula for ABA content determination was calculated, and the ABA contents of the samples were obtained by substituting the spectrophotometric values of each sample into the formula obtained using standard curve equation.

## Supplementary Material

Web_Material_uhab074

## Data Availability

All data included in this study are publicly available. All experimental data are provided in the attachment. The resequencing of 163 germplasm accessions where used from previous study published by our group, 34 accessions were resequenced for current study [4]; they have been uploaded to public databases or can be obtained by contacting the authors.
