# Supplementary material for: Genome-wide association analysis provides molecular insights into natural variation in watermelon seed size
Source: Hortic Res. 2022 Jan 19;9:uhab074. doi: 10.1093/hr/uhab074 (PMC8923815; doi:10.1093/hr/uhab074)
Supplement: Web_Material_uhab074 [file web_material_uhab074.zip › Supplementary figures.docx]

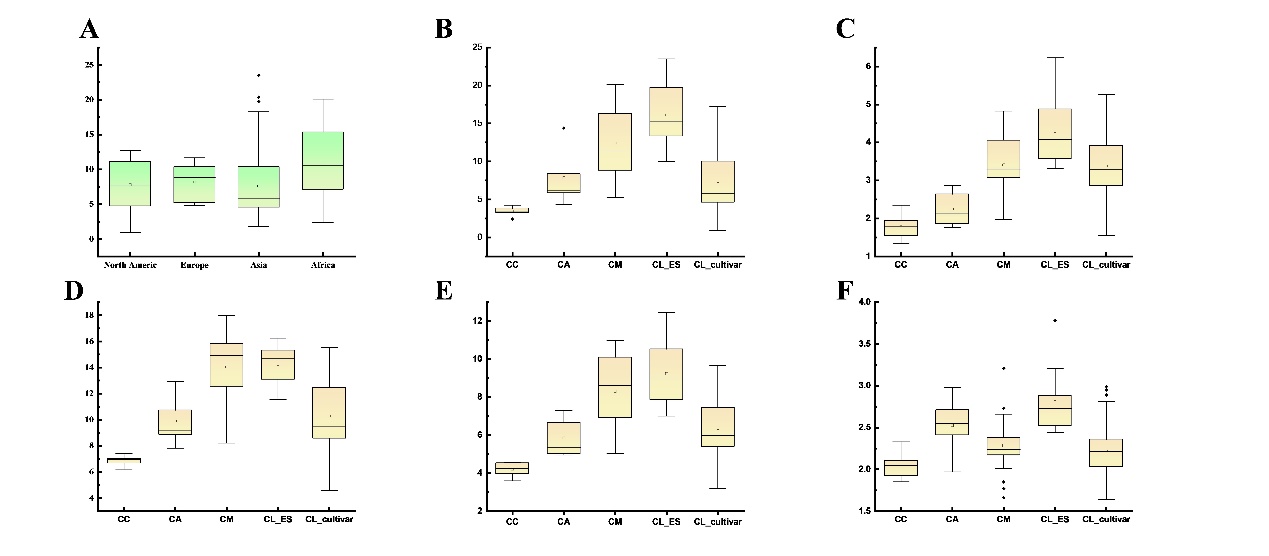


**Fig. S1** Statistical analysis of different types of watermelon in 2019. (**A**) The box plot of 100 SWT in different geographic origin watermelons (**B-F**) The box plot of 100 seeds weight (g), seed hilum width, seed length (mm), seed width (mm), seed thickness (mm) in different types watermelon, respectively CC: C. colocynthis; CA: *C. amarus*; CM: *C. mucosospermus*; CL_ES: *C. lanatus* edible seed watermelon; CL: *C. lanatus*.


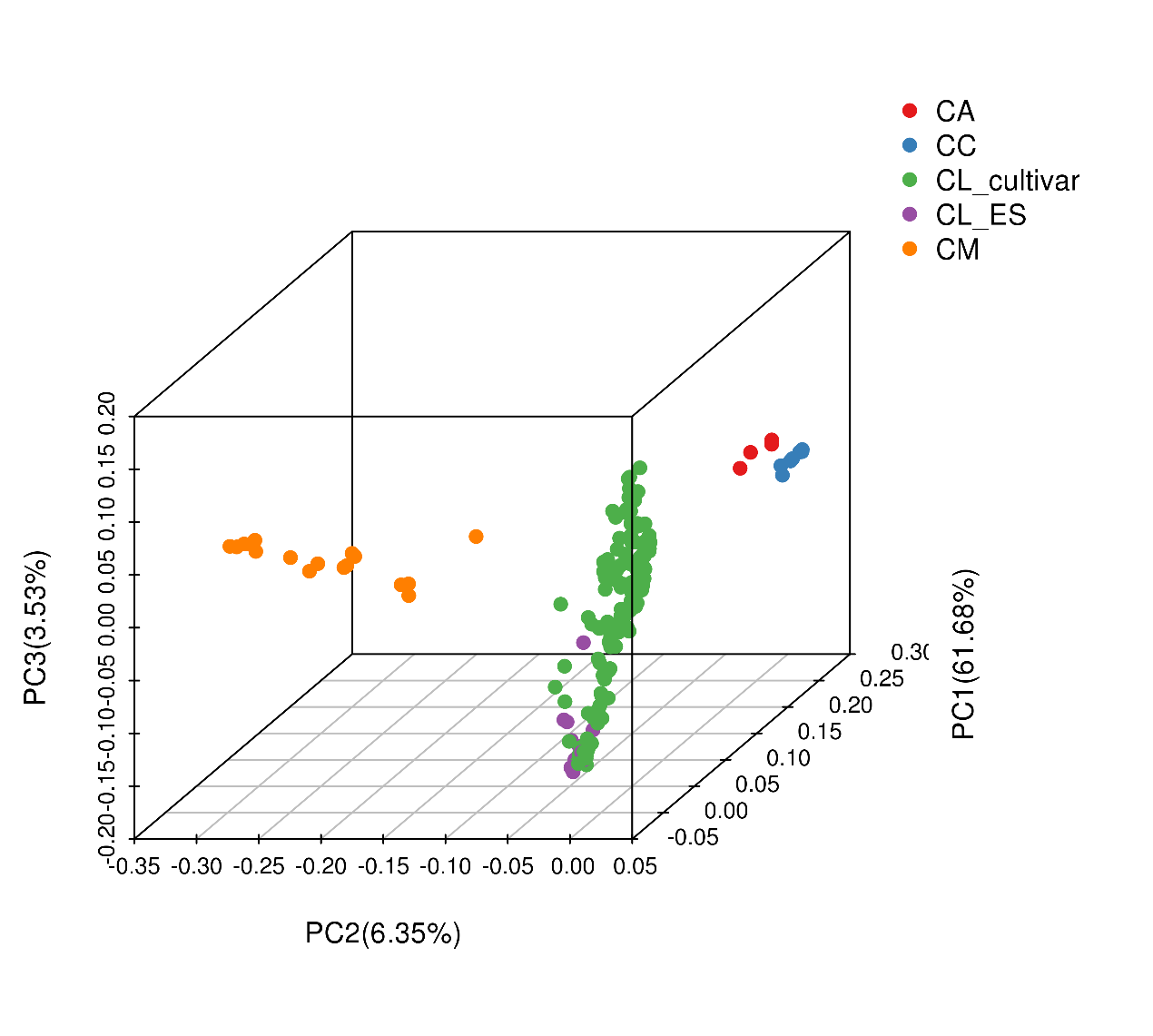


**Fig. S2** PCA plots of the first three components for the 197 watermelon accessions based on SNP data.


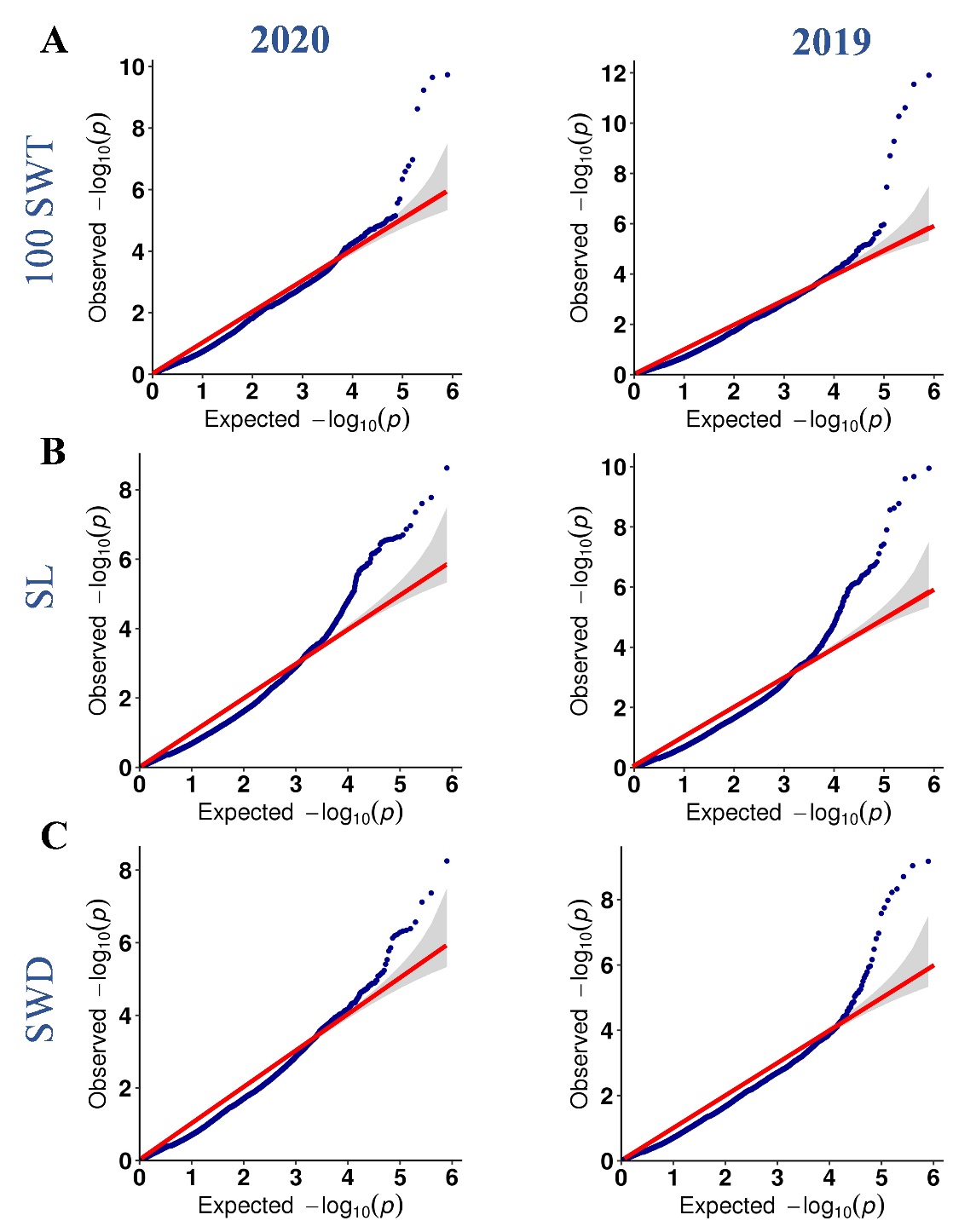


**Fig. S3** QQ plots of three seed traits, corresponding to the Manhattan plots of seed traits in Fig. 3. The abscissa represents the expected value, the ordinate represents the observed value, and the gray area represents the 95% confidence interval of the scatter points on the graph.


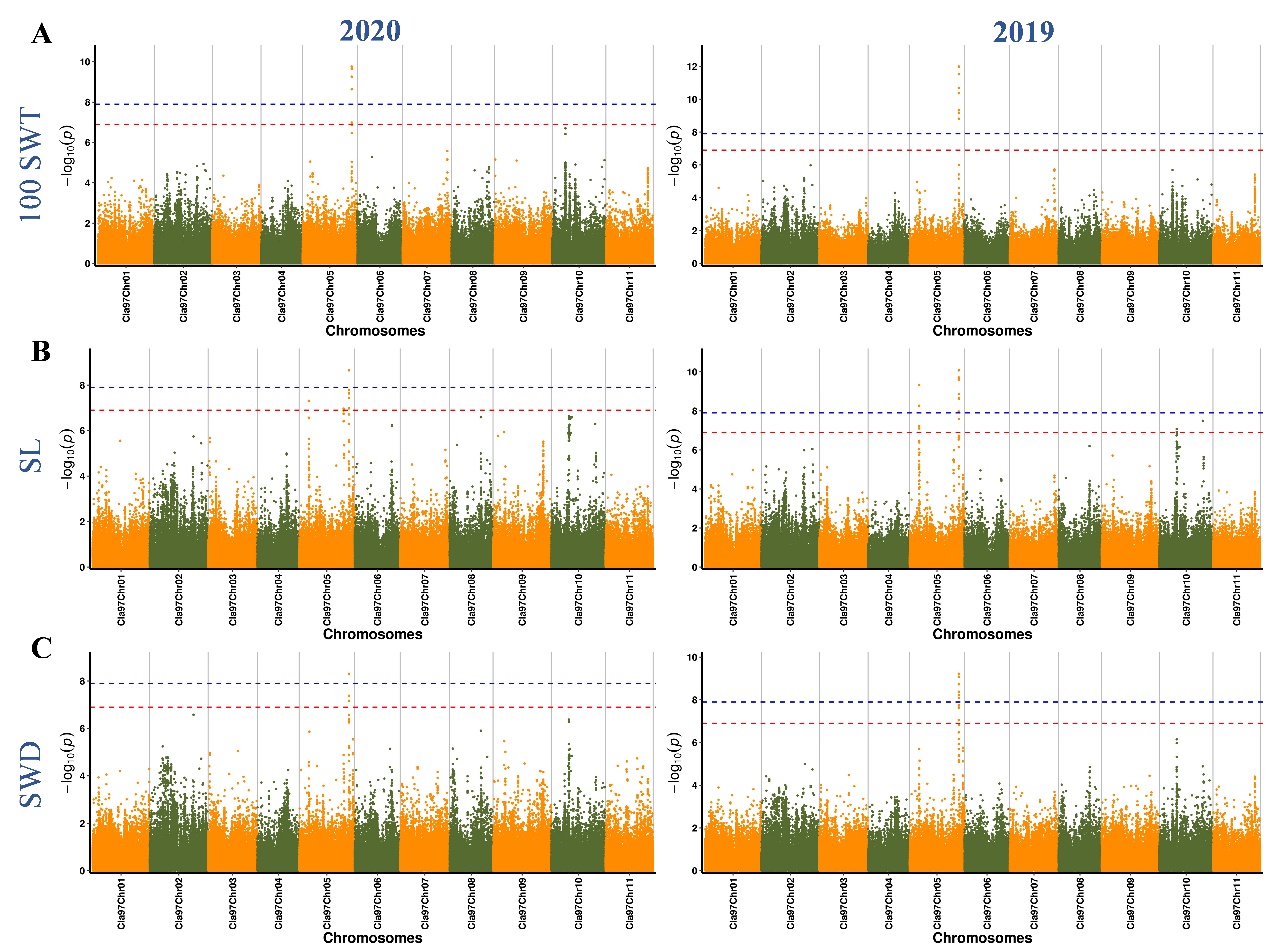


**Fig. S4** Manhattan plots of three seed size traits in 2020 and 2019 obtained by FAST-LMM algorithm.


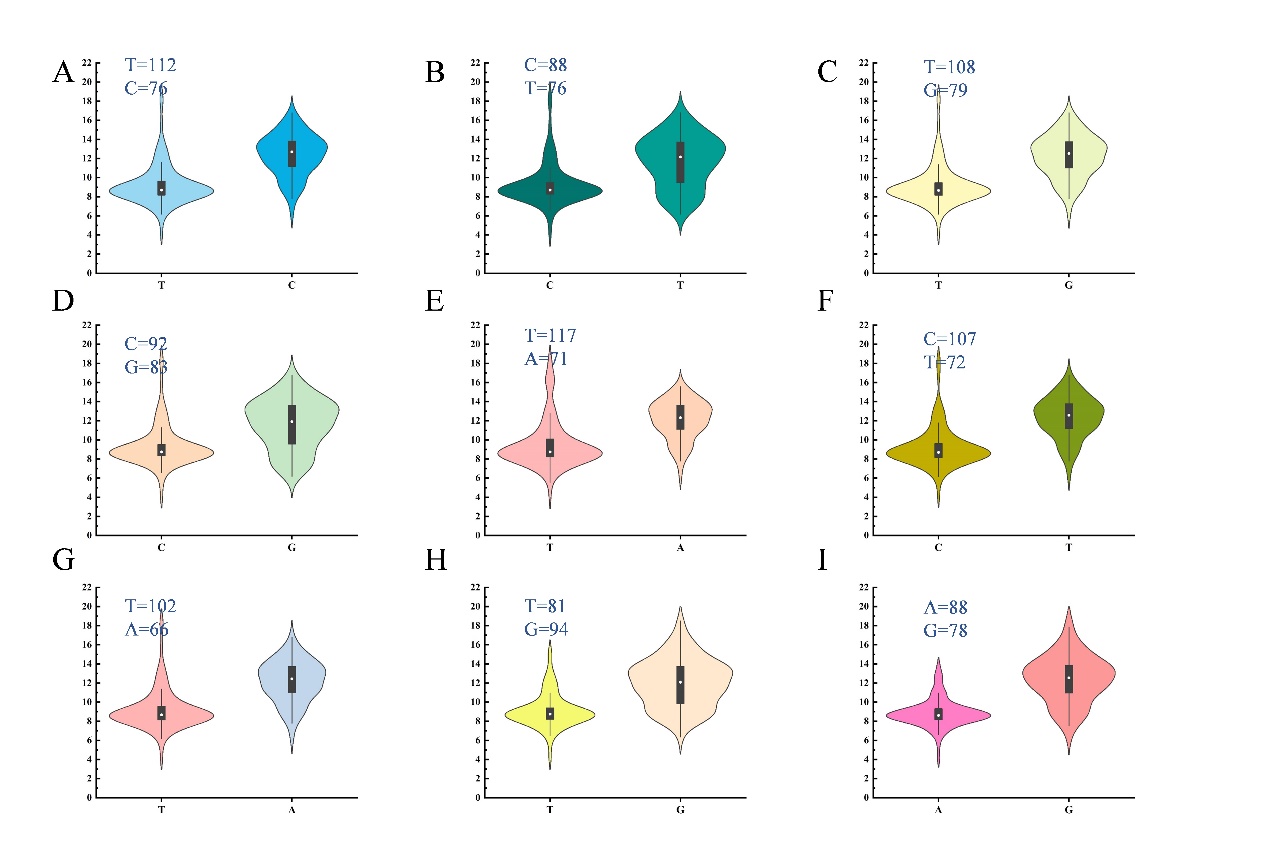


**Fig. S5** Violin diagram of significant SNP loci at different bases of seed length in 2020. Correspond to the description in Fig. 4.


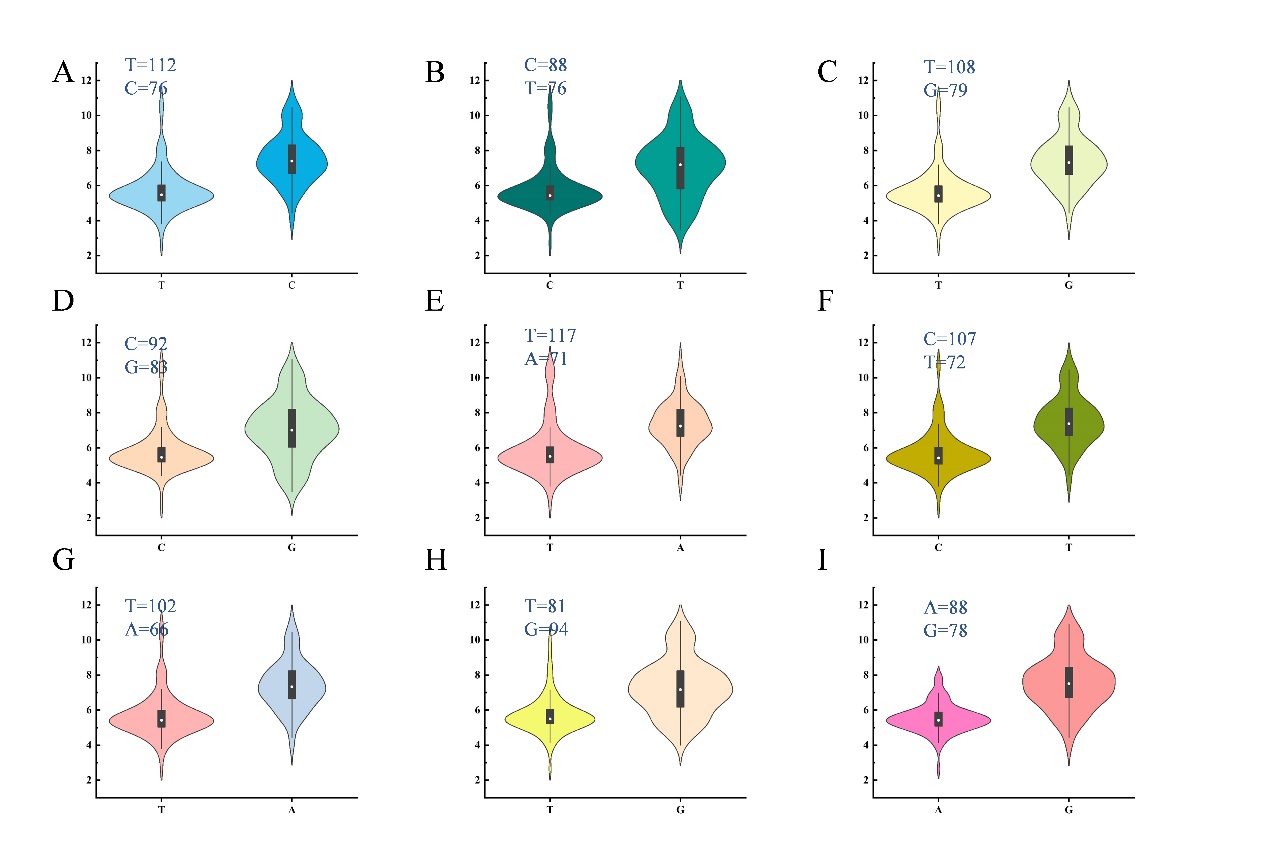


**Fig. S6** Violin diagram of significant SNP loci at different bases of seed width in 2020. Correspond to the description in Fig. 4.

**
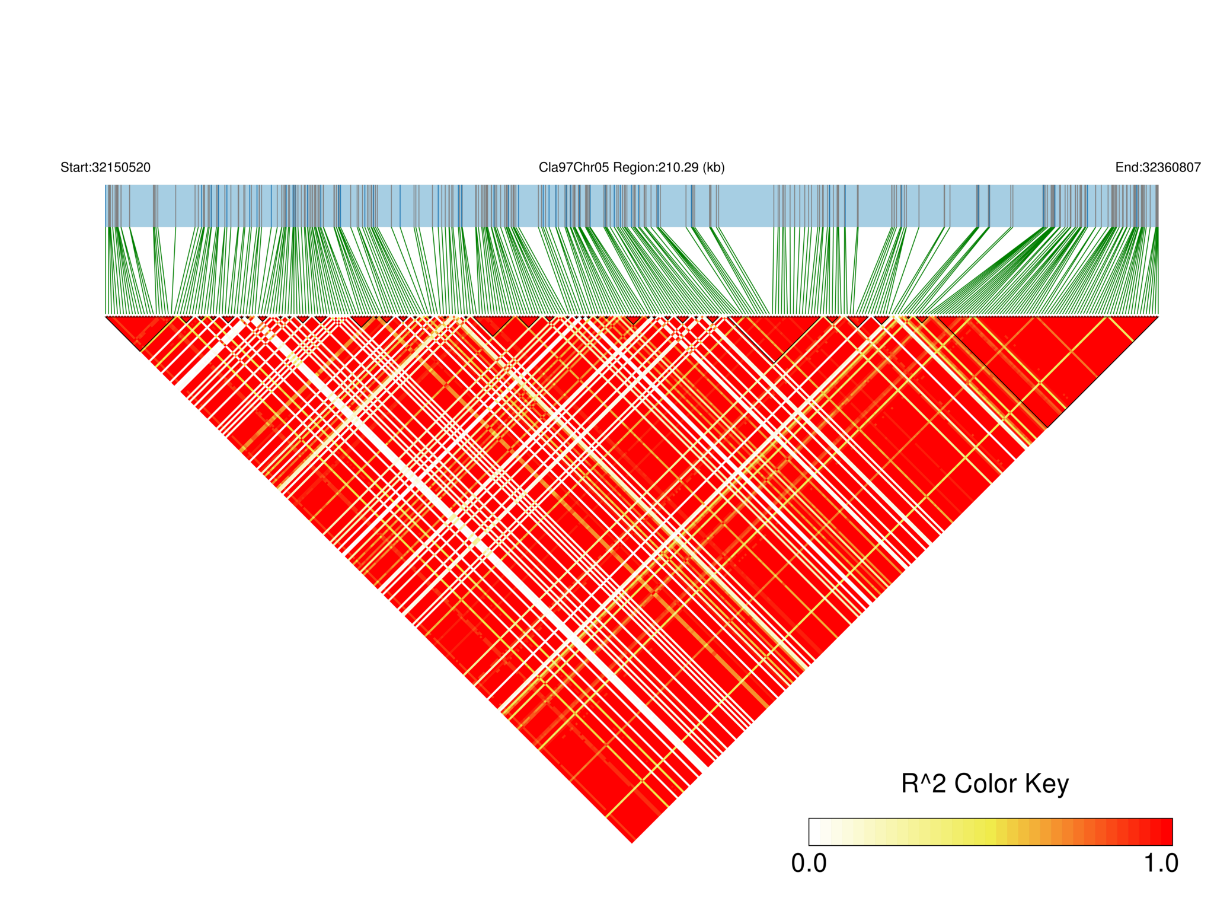
**

**Fig. S7** LD heatmap surrounding the peak on chromosome 5.

**
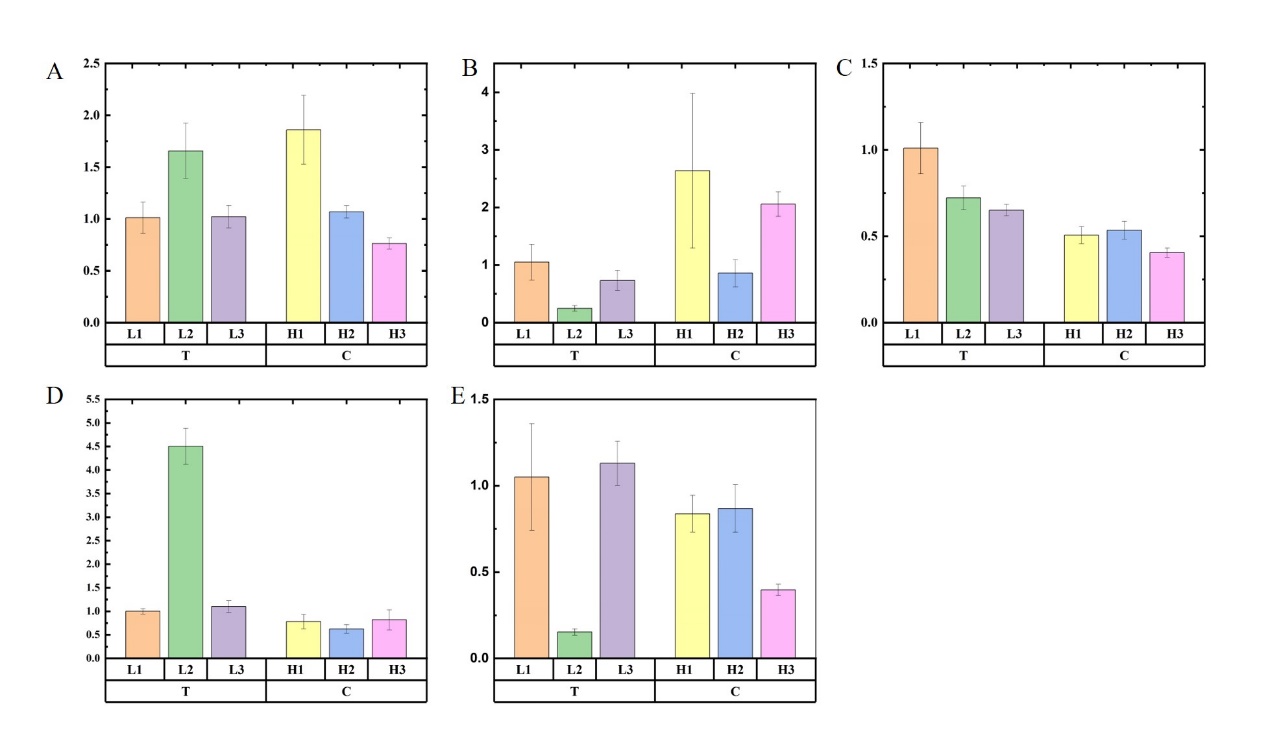
**

**Fig. S8** Gene expression levels of candidate genes at 20dap after pollination. (A-E) represent gene *Cla97C05G104340*, *Cla97C05G104350*, *Cla97C05G104360*, *Cla97C05G104380* and *Cla97C05G104390*. L1, L2, L3, H1, H2, H3 represent accessions XiangXiaoGua, SuXianXiaoZi, XiaoHongYu, HeTaoPi, JiZhuaGua and NingXiaHongZiGua, respectively. T and C represent the reference and mutant bases at SNP locus S5: 32250307 on chromosome 5.


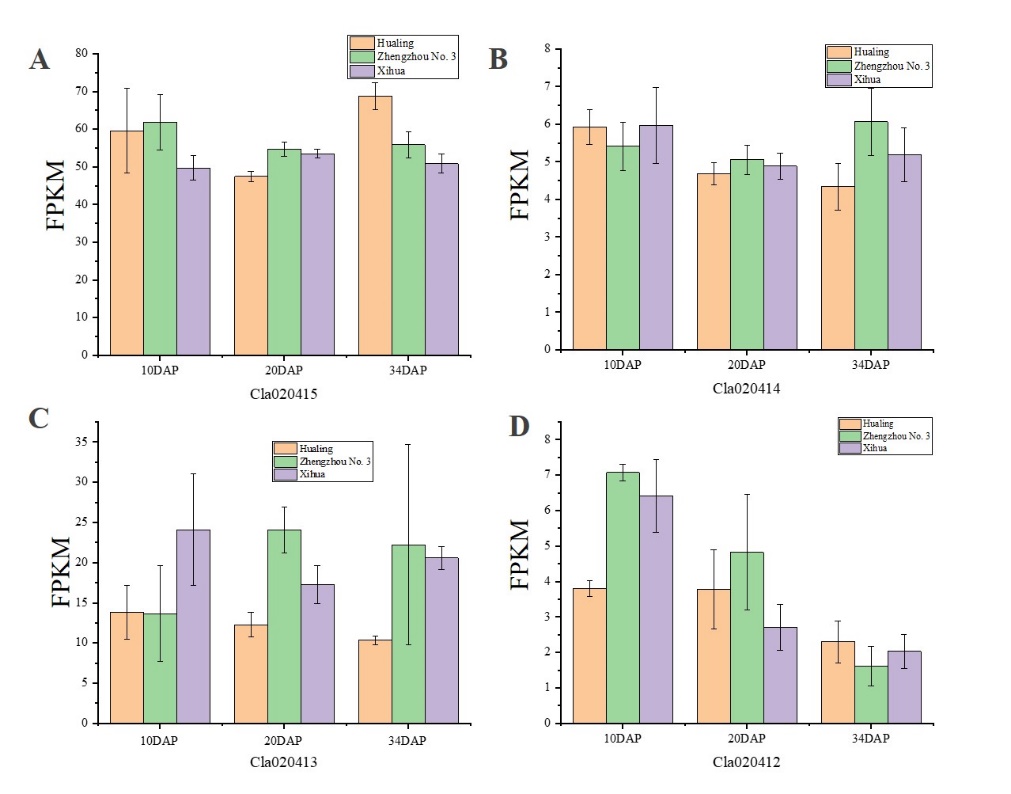


**Fig. S9** Gene expression levels of four candidate genes during watermelon flesh development. (A-D) Cla97C05G104340 (Cla020415), Cla97C05G104350 (Cla020414), Cla97C05G104360 (Cla020413), and Cla97C05G104380 (Cla020412). The average seed weight of Hualing was 9.13g, and the base of significant SNP S5: 32250307 was C. ZhengzhouNO.3 and Xihua have an average seed weight of 4.76g and 3.46g respectively, corresponding to the base T.
