# Supplementary material for: Genome-wide association analysis provides molecular insights into natural variation in watermelon seed size
Source: Hortic Res. 2022 Jan 19;9:uhab074. doi: 10.1093/hr/uhab074 (PMC8923815; doi:10.1093/hr/uhab074)
Supplement: Web_Material_uhab074 [file web_material_uhab074.zip › Supplementary tables.docx]

**Table S1** The information of 197 watermelon accessions and the data of five seed trait in 2020 and 2019.

**Table S2** Significant SNP information analyzed by GWAS

**Table S3:** The bases of nine significant SNPS on chromosome 5

**Fig. S4** qRT-PCR primers for 5 candidate genes
